# A Case of Retrocession of the Cutaneous Efflorescence in Rubeola

**Published:** 1851-09

**Authors:** C. H. Spilman


*A Case of Retrocession of the Cutaneous Efflorescence in Rubeola.* By C. H. Spilman, M. D.—On the 14th of July, 1850, Dr. James M. Singleton, of this place, was suddenly attacked with symptoms pathognomonic of measles. On the 17th, the characteristic eruption became visible, which, by the day following was fully developed. On the 3rd day of the eruptive stage, he left his room whilst the efflorescence was in full blast: passed about considerably in the open air, subjected himself to a sufficient amount of physical exertion to occasion some fatigue, resulting in an abrupt subsidence of the cutaneous eruption. His rapid convalescence for the time, however, quieted all apprehensions of serious injury from the sudden suppression. From that time until January following, a period of near six months, he enjoyed his usual health, with the exception of slight paucity of urine, together with a turbid furfuraceous appearance of this secretion. This latter quality, however, was not visible in the recent state. It seemed to possess, to some extent, the property of coagulability upon standing. As there was no accompanying pain, nor any other appreciable deviation from health, it attracted, at the time, but little attention.

About the middle of January last, whilst in attendance upon a course of lectures in the Kentucky School of Medicine, our young friend, as we are informed, after having suffered much from constipation of the bowels, and symptoms simulating a common cold for two or three weeks, was attacked with hectic fever, a dry hacking cough, and copious night sweats, exhibiting a well marked case of incipient *phthisis*.

Greatly emaciated from disease, which had been gradually progressive, he arrived at Harrodsburg on the 9th of March. Acquainted with the previous history of the case, we had a lurking suspicion that the repelled eruption had some agency in the production of the malady. Still, it seemed unaccountable, why the mal-development should have been so long delayed.

As this has been to us a novel and exceedingly interesting case, and as far as we are advised, has not a parallel in the annals of medical science, we will give a brief abstract of its features, progress, and treatment, from the time we took it in charge.

March 20th. Pulse 100. Respiration 25. Tongue slightly coated, rather red. Pain under the right axilla, extending round to the inferior margin of the scapula, increased on respiration. Constriction across the chest. Frequent dry hacking cough, with thirst and febrile exacerbation during the evening, terminating in the latter part of the night in a copious exhausting sweat, and free expectoration, sometimes mucous, but generally of a white, thin, homogeneous character: urine scant and turbid: a heavy dull pain, especially during the apyrexia in the sacral region.

*Auscultation* of the clavicular region detected a strong respiratory murmur, rather cavernous, mingled at times with a dry crepitant sound, and at others a gurgling ronchus. Percussion dull under each clavicle, more marked and extensive under the right. The inferior half of the left lung, sounds natural.

Directed:—Tinct. Sanguinariæ and Tinct. Iod. Fer., each thirty drops three times *per diem,* and Sulph. Fer. and Aloes, each three grains at night. Pustulation with Ung. Tart. Ant. upon the anterior surface of the chest.—Diet, chiefly of unbolted wheat flour and milk, with fruits.

This treatment was continued until the middle of April, with but slight amendment, except in the condition of the bowels, which were now in a soluble state. The fever and night sweats, however, were unabating, and the cough persistent. The sanguinaria and Iod. Fer., were now discontinued, and the Sulph. Quinia given with a view to control the paroxysms, and check the exhausting cutaneous evacuations. The quinia mitigated, but did not entirely control the paroxysms. The cough, however, became more frequent; the pleuritic stitches about the chest rather increased, and the expectoration greatly diminished under its use: and auscultation detecting a crepitant *rale,* which had not previously been so audible, the remedy after the fourth day was withdrawn. The iodide of iron being objected to in consequence of its unpleasantness, the myrrh mixture was substituted twice per day, and the aloe and iron pill at night. The stomach, however, did not tolerate the myrrh mixture which was on the 20th discontinued. The sanguinaria was now resumed in doses of thirty drops three times *per diem:* and with a view to control the hectic, the quinine and lactate of iron, two grains of each were given at intervals of three hours, commencing simultaneously with the sweat, and continuing until the paroxysm. The treatment was persevered in until the morning of the 27th, when, judge of our surprise, at finding our patient beautifully peppered over with the eruption of measles, upward of nine months from the date of its abrupt disappearance. The efflorescence was full and uniform: as complete and characteristic as any case we ever witnessed: remaining about the usual period, gradually receding, and, unlike the first appearance, was succeeded by distinct desquamation. But for the ravages the disease had made upon the lungs, as clearly evidenced by auscultation, we should have cherished some hope of a favorable issue of the case, as the explosion might be presumed, a triumph of the *vis medicatrix naturæ* over the power of the disease. The disorganized state of the lungs, and the exhausted condition of the general system, however, forbade such hope.

Without detaining the reader with the subsequent history of this case, we will simply remark, that the wasting diarrhea, so frequently an attendant of the last stage of *phthisis*, speedily supervened, during which the treatment was necessarily, only palliative, and he died on the 4th of June.

There are two or three inquiries, naturally suggested to our mind by this case which may be of practical importance.

1st. Whether or not the repelled cutaneous eruption had any agency in producing the disease in question?

2d. What the particular character of the morbid action during the latent period, prior to the second incubation?

3d. Would an earlier consummation of the rubeolous malady have been practicable; and had it been effected previous to the pulmonary lesion, would it not probably have saved the patient?—*Trans. Med. Jour., Aug.*

